# In Situ Inactivation of Selected *Bacillus* Strains in Brewer’s Spent Grain during Fermentation by *Lactococcus lactis* ATCC 11454—The Possibility of Post-Production Residues Management

**DOI:** 10.3390/foods12122279

**Published:** 2023-06-06

**Authors:** Patryk Pokorski, Monika Trząskowska

**Affiliations:** 1 Faculty of Human Nutrition, Warsaw University of Life Sciences (WULS), Nowoursynowska 159C, 02-776 Warsaw, Poland; 2 Department of Food Gastronomy and Food Hygiene, Institute of Human Nutrition Sciences, Warsaw University of Life Sciences (WULS), Nowoursynowska 159C, 02-776 Warsaw, Poland

**Keywords:** *Bacillus*, barley, brewer’s spent grain, biocontrol, foodborne pathogen, *Lactococcus lactis* ATCC 11454

## Abstract

The safety and quality of post-production residues is essential before they can be reused. Both to explore the possibility of reuse as a fermentation medium and the context of pathogens’ inactivation, the research aimed to characterize the fermentation system of *L. lactis* ATCC 11454 and brewer’s spent grain, malt and barley, especially to in situ inactivation of selected *Bacillus* strains during the fermentation and storage. Barley products were milled, autoclaved, hydrated and fermented with *L. lactis* ATCC 11454. Then, the co-fermentation with *Bacillus* strains was carried out. The amount of polyphenols in the samples ranged from 483.5 to 718.4 ug GAE g^−1^ and increased after 24 h fermentation with *L. lactis* ATCC 11454. The high viability of LAB in the fermented samples and after 7 days of storage at 4 °C (8 log CFU g^−1^) indicates the high nutrients bioavailability during the storage. Also, this co-fermentation on different barley products indicated a high reduction level (2 to 4 logs) of *Bacillus* due to the biosuppression effect of the LAB strain in this fermentation system. Brewer’s spent grain (BSG) fermented with *L. lactis* ATCC 25 11454 produces a highly effective cell-free supernatant (CFS) for suppressing *Bacillus* strains. This was evident in both the inhibition zone and fluorescence analysis of bacteria viability. In conclusion, the obtained results justify the use of brewer’s spent grain in selected food products, increasing their safety and nutritional value. This finding is highly beneficial in the sustainable management of post-production residues when current waste material can still serve as a source of food.

## 1. Introduction

Consumers are becoming more aware of the environmental importance of the food production process as well as the impact of food quality on their health [1]. Therefore, they choose foods produced according to the sustainable development strategy: Reduced water waste and greenhouse gas emissions during food production [2,3,4] and health-promoting products, i.e., functional, fortified and/or not/less processed [5,6]. They are also aware that the agriculture or food industry sector produces an excess of post-production waste, hence they are more and more open to food produced with the use of post-production raw materials [7]. Food design with the use of post-production raw materials is currently developing rapidly and concerns both post-production residue plant-origin [8,9,10] as well as animal-origin [11,12]. This is in line with the current zero waste or less waste trend [13]. One of the post-production wastes, which has so far been used usually for feeding animals rather than for human consumption, is brewer’s spent grain–residue from the brewing industry in the production of beer. Its production volume is estimated at 20 kg BSG per 100 L of beer produced; thus, it is estimated that the BSG worldwide annual production exceeds 300 million tons [14]. Recently, its high nutritional value, i.e., high content of protein (10–17% dry matter (dm)), the content of β-glucan (4–9% dm) or dietary fiber (11–13% dm) has been noticed and BSG has been used in the industry for the production of bread, confectionery products and other bakery products as well as snack-type products [15,16]. When applied to the production of fermented milk drinks, BSG was able to shorten fermentation time (by increasing the optimal condition of fermentation and nutrients) and prevent syneresis [17]. Application of BSG into fermentation medium was also associated with the extended ability to survive LAB during storage of the number above 6 log CFU mL^−1^ of products which is one of the most important factors to qualify any products as probiotics [18]. 

Contamination of food products with *Bacillus cereus* is estimated to be responsible for 1.4–12% of all food poisoning outbreaks worldwide. Moreover, two types of gastrointestinal diseases caused by toxin cereulide, such as emesis and diarrhoea, have been described. Consumption of bacteria is not necessary although it was generally thought that at least log 3 to log 5 CFU *B. cereus* per g of food was needed to produce cereulide in disease-provoking concentrations [19]. The comprehensive analysis conducted by Messelhausser [20] includes more than 3500 food samples, indicating that *B. cereus* was detected in 5% of them and the grain-based products have been qualified as a high-risk category of food. *Bacillus subtilis* has not been considered as human pathogen although some strains of this species may occasionally cause food poisoning [21]. Additionally, *B. subtilis* has been characterized by high resistance to conventional decontamination methods and could figure even in high-processed food products [22]. The amount of bacterial contamination of grains is usually low (approx. log 2 CFU g^−1^) but occurs frequently due to native environmental contamination and/or human involvement, as well as transport and storage conditions [23]. Conventional decontamination methods in grains included dehulling, temperature inactivation as well as chemical disinfection with hydrogen peroxide and lactic acid. On the other hand, novel techniques involve cold plasma, irradiation, ozone and pulse light [24]. Both of the techniques, although they show many advantages, such as high efficiency and precise operation, also have some limitations as invasiveness and/or change of properties (conventional techniques) or are used relatively rarely for reasons of the financial aspect (novel techniques). The development and implementation of new methods that are characterized by high efficiency of inactivation of pathogenic microflora, and at the same time may bring additional health benefits to consumers, is an important and promising direction for sustainable development understood as a reliable and safe food source. Using the naturally occurring potential of LAB to reduce the number of foodborne pathogens is in the line and trends nowadays [25,26]. Co-fermentation with LAB and metabolites production is considered a good bio-preservative agent due to their non-toxic, non-immunogenic and thermo-resistance characteristics [27].

*Lactococcus lactis* spp. *lactis* is classified as lactic acid bacteria (LAB), and its numerous healthy properties, such as lowering blood pressure, improving lipid profile and glucose metabolism, and anti-obesity effects, were documented in previous research [28]. Moreover, *L. lactis* has been found to have the most comprehensive recognition for its antimicrobial activity thanks to its ability for bacteriocin secretion [29]. Nisin, produced by *L. lactis* subsp. *lactis*, is generally recognized as a safe (GRAS) bacteriocin, which has been approved as a food additive by both the Food and Drug Administration (FDA) and the European Food Safety Authority [30,31]. Studies aiming to determine the ability of the *L. lactis* to reduce a number of pathogenic bacteria were carried out only in the narrow spectrum of strains, i.e., *Streptococcus iniae* KCTC 3657, *Staphylococcus aureus* subsp. *aureus* KCTC 1928, *Listeria monocytogenes* KCTC 13064, among others [30]. A wider characterization of antimicrobial activity among several strains of one of the species was not performed until now.

The antimicrobial properties of several plant-derived raw materials containing simple phenolic acids, polyphenols and terpenes have been described [32,33,34]. Moreover, pure oligosaccharides, such as β-glucans and β-glucooligosaccharides, were recognized as a bacteriostatic factor relative to pathogens from the family *Bacillaceae*, *Listariaceae* and *Enterobacteriaceae* [30,35]. When these oligosaccharides were used in the fermentation process with *Lactococcus lactis* subsp. *Lactis*, an increase of nisin secretion by over 25% was observed [30]. The conducted research was based only on the addition of pure oligosaccharides as a potential prebiotic into the medium [30]. What is more, the ability of the system of *L. lactis* subsp. *lactis* and barley products in different forms of processing and nutrient availability as grain (not processed), malt (germinated) and BSG (mashed) in the context of maintaining food safety were not described yet. Both to explore the possibility of reuse as a fermentation medium and the context of pathogens inactivation, the research aimed to characterize the fermentation system of *L. lactis* ATCC 11454 and brewer’s spent grain, malt as well as barley, especially to in situ inactivation of selected *Bacillus cereus* and *Bacillus subtilis* strains during the fermentation process and storage. The analysis of barley products included the composition of oligosaccharides and the total content of polyphenols. 

## 2. Materials and Methods

**Figure 1:**
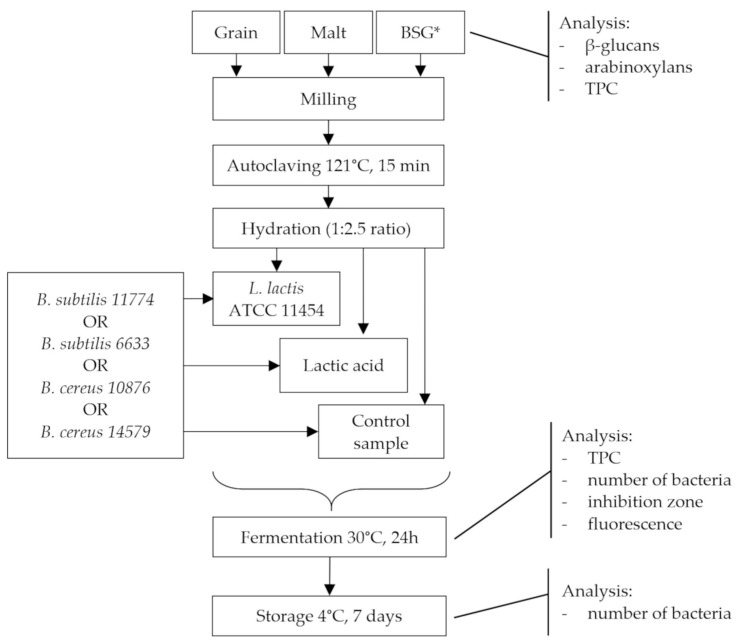
The diagram of the experiment. Explanation: BSG*—brewer’s spent grain.

The diagram of the experiments is presented in Figure 1. Barley products (BP) were milled, autoclaved at 121 °C for 15 min, weighed in the 50 mL Falcon tubes and hydrated. In general, to obtain 10 g of the samples, 2.86 g of BP and 7.14 mL of sterile water were used (1:2.5 ratio). The hydrated sample was inoculated with 100 µL of *L. lactis* ATCC 11454 containing approximately 8 log CFU mL^−1^ and vortexed at 3200 rmp. The fermentation process was carried out at 30 °C for 24 h. Sterile working conditions were maintained during each sampling of the analysis by performing determinations in a Class II Biohazard Safety Cabinet with constant airflow at 22 °C (ESCO^®^, Horsham, PA, USA).

### 2.1. Raw Materials

To assess the influence of processing three barley products, barley grains from organic farming (Batom, Kraków, Poland), Pale Ale barley malt (WikingMalt, Strzegom, Poland) and brewer’s spent grain were used. BSG was produced according to Neffe-Skocińska et al. [36] with modification as follows: One kilogram of the malt was used for 3 L of tap water to mash. The malt was mashed using a single infusion method for one hour at 68 °C in an automatic brewery kettle Coobra CB3 PRO (CBF Drinkit; Möltand, Sweden). The mash was then heated to 80 °C and held at that temperature for 15 min to inactivate enzymes. Then, the wort was filtered and the BSG was sparged with water at 90 °C. The filter bed from spent grain was washed until the extract concentration in the obtained liquid was 2 g 100 mL^−1^. Measurement of the extract concentration (soluble sugars, acids and other compounds) was made by the refractometer (Silverando Co., Beijing, China). 

### 2.2. Bacterial Strains 

The *Bacillus* strains from a microbial collection of Department of Food Gastronomy and Food Hygiene, Institute of Human Nutrition Sciences, SGGW-WULS were used as follows: *Bacillus cereus* ATCC 10876, *Bacillus cereus* ATCC 14579, *Bacillus subtilis* ATCC 11774 and *Bacillus subtilis* ATCC 6633 were activated from the lyophilized pellet by spreading on the selective medium such as Polymyxin Pyruvate Egg yolk Mannitol Bromothymolblue Agar (Neogen^®^ Culture Media, Lansing, MI, USA). Cultivation was conducted at 37 °C for 24 h, then a single colony of each strain was transferred to 10 mL of Brain Heart Infusion broth (Merck, Poznań, Poland) and was incubated at 37 °C for 24 h to achieve approx. 8 log CFU mL^−1^ of bacteria. *L. lactis* ATCC 11454 was used as an indicator strain and was activated from a lyophilized pellet by spreading on the M17 Agar (Biolab, Budapest, Hungary) and cultivated at 30 °C for 24 h. Then, a single colony was transferred to 10 mL of MRS Broth (Biomaxima, Lublin, Poland) and was incubated at 30 °C for 24 h to achieve approx. 9 log CFU mL^−1^ of bacteria. Suspension of all strains was centrifuged at 10,000× *g* rpm for 10 min and the liquid media was two times replaced with Phosphate buffered saline (Sigma-Aldrich, St. Luis, MO, USA). 

### 2.3. Moisture and β-Glucans Content Determination 

Barley grain moisture was determined by the gravimetric method after drying at 105–107 °C, according to PN-R-74110:1998 [37]. The moisture content of the BSG was determined by the gravimetric method after drying according to 12.2 Analytica EBC [38]. Malt moisture was determined by the gravimetric method after drying based on PN-A-79083-5:1998 [39]. β-glucan content was determined by spectrophotometry after enzymatic hydrolysis according to 995.16 AOAC, 32-23 AACC, ICC Standard No. 166 (the method is specific to [(1–3)(1–4)]-β-D-glucan) [40]. 

### 2.4. Arabinoxylans (AX) Content Determination 

The content of non-starch polysaccharides (NSP) was determined by gas chromatography (GC) according to Englyst and Cummings [41] and AACC standard procedure 32–25, AOAC 994.13. The NSP content was calculated as the sum of the sugars: arabinose, xylose, mannose, galactose and glucose (Approved Methods of the AACC, 2003; AOAC, 1990). This analysis allowed the separation of NSP into two fractions: soluble (S-NSP) and insoluble (I-NSP), and the determination of the composition of polysaccharides in both fractions. The arabinoxylan content of each fraction was calculated as the sum of arabinose and xylose. 

Enzymatic hydrolysis of starch is carried out using α-amylase and amyloglucosidase enzymes (Neogen). Then the samples were centrifuged and separated with 96% ethyl alcohol (Chempur, Piekary Śląskie, Poland) into soluble (supernatant) and insoluble (pellet) fractions. Each fraction was hydrolyzed with 1 M sulfuric acid (100 °C, 2 h) to monosaccharides and then converted to volatile alditol acetates. Samples prepared in this way were separated on a Clarus 500 gas chromatograph (Palo Alto, CA, USA) equipped with an Rtx-225 quartz capillary column (0.53 × 30 m), autosampler, split injector and flame ionization detector (FID). The carrier gas for analysis was helium. The separation was carried out at a temperature of 225 °C, with the temperature of the injector and detector at 275 °C. 

### 2.5. Total Polyphenols Content (TPC) Determination

The TPC was determined both in the raw material (before fermentation) and in fermented samples. To determine the total polyphenols content, the method carried out by Niroula et al. [42] was adopted with modification. Briefly, the extraction was carried out with 100 mg of the sample diluted in 5.0 mL of solvent 80% (*v*/*v*) methanol (Chempur). Ultrasounds-assisted extraction (UAE) with a controlled cycle (15 min, 40 °C, 550 Hz) has been applied. Then, the solution was centrifugated for 10 min 10,000× *g* rmp and the supernatant was collected for further analysis. TPC was determined spectrophotometrically by using Folin-Ciocalteu’s reagent (Chempur, Piekary Śląskie, Poland). 20 µL of the sample was poured into a 96-well plate (NEST, Wuxi, China), and 100 µL of the Folin-Ciocalteu’s reagent was added. The samples were incubated for 5 min in a dark place. 

Then, 80 µL of sodium carbonate (Chempur) dissolved in deionized water (7.5 % *w*/*v*) was added, gently mixed and incubated for another 2 h. The absorbance was measured at 750 nm in a SpectraMax Id3 Multi-mode Microplate Reader (San Jose, CA, USA). TPCs were evaluated as gallic acid equivalent (GAE) by using a typical calibration curve (R2 ≥ 0.99) prepared with gallic acid as standard (0.01–0.1 mg mL^−1^). The results expressed as a mean standard deviation (*n* = 5), 3 repetitions for each trial were performed.

### 2.6. Antimicrobial Properties and Survival of Bacteria

The experiment was performed in 3 variants as follows: (I) 10 g of the sample mixed with 100 µL of the *Bacillus* strains suspension containing approx. 10^7^ CFU mL^−1^ (control sample); (II) 10 g of the sample mixed with 100µL of the *Bacillus* strains (approx. 7 log CFU mL^−1^) and 20% lactic acid added after 9, 18 and 24 h of the fermentation to maintain a pH as in variant III; (III) 10 g of the samples mixed with 100 µL of the *Bacillus* strains (approx. 7 log CFU mL^−1^) and 100 µL of the *L. lactic* ATCC 11454 (approx. 8 log CFU mL^−1^). 

The co-fermentation was performed at 30 °C for 24 h. After this stage, the number of bacterial cells was determined by the spread plate technique twice: Firstly, immediately after the fermentation (0 days) and for a maximum of one week at 4 °C (7 days). Appropriate decimal dilution of the samples was applied to the Petri dishes with the solidified Mueller-Hinton agar (I, II, III variant) and MRS agar (III variant) and incubated for 24 h at 37 °C. After incubation, typical colonies were counted. For each trial, 3 repetitions have been made. 

#### 2.6.1. Inhibition Zone

The ability of a bacteriocin-like inhibitory substance (BLIS) in the form of cell-free supernatant (CFS) of fermented barley products to inhibit the growth of selected *Bacillus* strains was investigated using the Agar disk-diffusion method based on Lalpuria et al. [43].

The bioassay of 15 mL Mueller-Hinton Agar (Biomaxima, Lublin, Poland), with a 1% addition of Tween 20 (Merck, Poznań, Poland) as a surfactant, was aseptically poured into sterile Petri dishes. Into solidified medium 100µL of each *Bacillus* suspension containing approximately 10^5^ CFU mL^−1^ was spread and left to absorb. The disk Ø 5 mm was dried at a temperature of 150 °C for 1 h in a convection oven (Rational AG, Landsberg am Lech, Germany). To obtain CFS, the fermented barley products were centrifuged twice. Firstly at 5000 rpm for 15 min to separate the pellet and supernatant. Then, the suspension was transferred into 2 mL plastic Eppendorf and centrifuged at 10,000 for 15 min to separate the *Lactococcus lactis* cells. The cell-free supernatant was collected for further analysis. Each disk was immersed in the CFS, transferred into Petri dishes and allowed to absorb. In one Petri dish, 6 disks were placed. The mean volume of absorbed CSF by each disk was estimated as 6.5 ± 0.1 µL (*n* = 20). The pre-diffusion was conducted for 48 h at 4 °C; then, the Petri dishes were incubated overnight at 37 °C. The inhibition zone was measured horizontally using ProtoCOL3 (Synbiosis, Cambridge, UK). The preparation of the standard curve individually for each strain of *Bacillus* was adopted from Thanjavur et al. [44]. Nisin A from *Lactococcus lactis* (2.5% in purity) was purchased from Sigma-Aldrich, and different concentration of nisin (40, 200, 600, 1000, 2000 IU mL^−1^) was employed. The experiments were performed in triplicate.

#### 2.6.2. LIVE/DEAD Fluorescence Assay of Bacterial Viability 

A LIVE/DEAD BacLight kit containing SYTO-9 and propidium iodide dyes (Molecular Probes Inc., Waltham, MA, USA) was used to assess the survival of *Bacillus* strains in the environment of BLIS. SYTO-9 labels cells with both damaged and intact membranes, whereas propidium iodide penetrates only cells with damaged membranes. Thus, the ratio of the fluorescence emission between live bacterial cells (wavelength of 530 nm; green) and the dead bacterial cells (wavelength of 630 nm; red) can be estimated. 

In this experiment, the CFS from fermented BSG was used as an inhibition agent, due to the highest potential to inhibit the growth of *Bacillus* cells indicated in the previous experiments. Acquiring of cell-free supernatant has been described in the previous section. The ratio between the suspension of *Bacillus* cells and CFS as well as the duration of storage was adopted from the research of Thanjavur et al. [44] with modifications. Briefly, each of the *Bacillus* strains was suspended in sterile, deionized water. 50 µL suspension of the *Bacillus* cells (approx. 10^6^ CFU mL^−1^ and 950 µL of the CFS) were used in the experiment. Then, the vials were stored for 24 h at 4 °C. After this stage, the samples were centrifuged at 10,000 rpm for 15 min, and the cell-free supernatant was replaced by sterile, deionized water. 100 µL of the bacterial suspension was transferred into a 96-well BRAND Pure Grade S plate with the clear bottom (Brand GMBH + CO KG, Wertheim, Germany) for fluorescent analysis. The procedure for staining and reading the fluorescence emission has been adopted from the protocol of LIVE/DEAD^®^ BacLight™ Bacterial Viability Kits and performed in a SpectraMax Id3 Multi-mode Microplate Reader. A standard curve was prepared with ratios of live and dead cells adopted from LIVE/DEAD^®^ BacLight™ protocol. To obtain dead cells, autoclaving at 121 °C for 15 min to prevent the formation of spores was investigated. The analyses were performed in 3 replications. 

### 2.7. Statistical Analysis 

Statistical analysis of the results was carried out using Statistica 13.3 (StatSoft, Kraków, Poland) as well as Microsoft Excel 2019 (Microsoft, Redmond, WA, USA). Mean and standard deviation was calculated. The homogeneity of variance and the normality of the distribution of results were checked, and variance analysis (ANOVA) or non-parametric equivalent (Kruskal-Wallis or U Mann-Whitney tests) were used accordingly. When applied, ANOVA was performed with Tukey’s post hoc test. Statistical significance was recognized when *p* < 0.05.

## 3. Results

### 3.1. Oligosaccharide Profile

The content of arabinoxylans and β-glucans is presented in Table 1. The amount of AX and β-glucans in the BP has been determined on average 10.72 g per 100 g and 1.89 g per 100, respectively. Generally, the impact of processing (germination, mashing) had a substantial effect on the profile of oligosaccharides. The more processed barley product, the higher content of total soluble arabinoxylans (S-AX), as well as the insoluble fraction of arabinoxylans (I-AX) and less content of β-glucan were observed. Recent research conducted on hydrolyzed barley β-glucans into β-GOS showed that they may be a bacteriostatic factor concerning foodborne pathogens [30]. Therefore, a profile of natively occurred oligosaccharides might determine the bacteriostatic properties of the fermented system and indeed had a synergistic effect on the produced LAB bacteriocins [30]. Izydorczyk & Dexter [45] and Zanninni et al. [46] declared the content of both oligosaccharides in a wider range: for AX from 1.5% to 16.4% and for β-glucans from 2.5% to 11.3%. Moreover, in the conducted research, the decreasing trend of the content of β-glucans due to the processing (malting and mashing) was observed (Table 1). Maintenance of the optimal condition, such as temperature, duration of the process, pH and substrate concentration, during the processing could activate the native enzymes, such as α-amylase, α-glucosidase, among others, which cause the hydrolysis of the long chains of polysaccharides to shorter ones, which was confirmed in this study. In the research of Chen et al. [47], it was highlighted that maintaining or enriching the fermentation medium with oligosaccharides as fructooligosaccharides and/or trehalose, among others, the secretion of nisin by *L. lactis* can be effectively increased. Endo [48] studied the effect of eight different oligosaccharides on LAB growth and metabolism. According to their results, β-GOS was characterized as those which caused the highest growth of LAB. In addition, it is noteworthy to declare that the identified oligosaccharides exhibit diverse prebiotic properties. Furthermore, through oligosaccharide profiling, the intriguing metabolic pathway in LAB strains was recognized. Thus, it can be assumed that increasing the prebiotic potential by using several sources (AX, β-GOS, β-glucans) will have a greater impact on the growth and viability of LAB as well as the volume of their metabolites production than single ones such as purified oligosaccharides. The naturally high content of the aforementioned oligosaccharides in BSG confers upon it the ability to stimulate the growth and viability of LAB during fermentation. These properties make BSG a valuable resource for promoting the growth of LAB and improving the overall fermentation process. From the food safety point of view, it is also justified because the positive association between the viable cells of nisin-producer strains and the volume of secreted nisin has been documented [49]. 

### 3.2. Total Polyphenol Content

In general, TPC in the studied raw materials differs significantly (*p* < 0.05). The highest polyphenols content was in barley malt, which contained 10% more polyphenols than BSG and almost 50% polyphenols more than barley grains (Table 2). It has been shown that fermentation increases the content of free forms of phenolic acids in barley, especially caffeic acid and ferulic acid, and increases their bioavailability [6,50,51]. While phenolic compounds are found in both free and bond forms in cereals, most of them are in bound form. When polyphenols fermented with LAB were transformed into free forms, thereby significantly increasing the content of the polyphenols in fermented products, which positively correlates with antioxidative activity [6,52]. In conducted research, the phenomenon of the relationship between TPC and degree of processing has also been observed. The fermentation process was positively associated with increasing the TPC in all of the samples, but the increase of TPC (1.4–4.1%) was not significant (*p* > 0.05).

Naturally occurring non-flavonoid polyphenols, including hydroxycinnamic acids as caffeic acids, and ferulic acids, have been found to inhibit foodborne pathogens such as *Bacillus cereus*, *S. aureus* and *P. fluorescens* [33]. The diverse mechanism of action of the polyphenols against foodborne pathogens via cell membrane rupture, defective nucleic acid mechanisms and decay of the proton motive force was studied previously [33]. A recent study conducted on polyphenols showed that their metabolism in the colon exhibited analogous effects as conventional prebiotics and could induce the growth of LAB as well as can support the potential of the other antimicrobial agents via synergistic effects [33,53].

### 3.3. Viability of Lactococcus Lactis ATCC 11454

A significant association between the level of BP processing and *L. lactis* ATCC11454 cell growth was observed (*p* < 0.05) (Table 3). The highest growth of the *L. lactis* ATCC 11454 was observed on the BSG as a medium, after fermentation and 7 days of storage. In the grain and malt samples, after the storage, a slight decrease in the number of *L. lactis* ATCC 11454 was observed in contrast to growth on BSG, although the change was not significant (*p* > 0.05).

It is worth noting that, in all of the samples, the intensive growth of the *L. lactis* ATCC 11454 was achieved (>10^6^ CFU/g). This phenomenon was also observed in the research conducted by Naibaho et al. [17]. The addition of 20% BSG was used to make fermented milk products and caused the significantly (*p* < 0.05) higher viability of *S. thermophilus* and *L. bulgaricus* as well as total LAB during 14 days of storage. In the research of Mitri [54], the addition of BSG was associated with the increasing ability of LAB to survive as well as shortening the fermentation process, reducing the syneresis of yogurt and maintaining the flow behavior of fermented milk drinks. The fermentation of BSG not only enhances the production of value-added components like amino acids, volatile fatty acids, enzymes, and vitamins but also maintains a stable production of lactic acid. This decrease in pH was positively correlated with higher viability of LAB [17].

### 3.4. Antimicrobial Activity

#### 3.4.1. Susceptibility of Strains and Impact of Barley Products 

A number reduction of selected *Bacillus* strains was recorded in all of the samples where *L. lactis* ATCC 11454 was co-fermented. *B. subtilis* 6633 was characterized by a highest sensitivity to antimicrobial activity in the co-fermentation experiments because their exposition to *L. lactis* ATCC 11454 was associated with the highest reduction ranging to 4 log CFU g^−1^. In contrast, the lowest reduction (2 log CFU g^−1^) was observed on the *B. subtilis* ATCC 11774. The medium used also played a role in the reducing effect on *Bacillus* cells. It was noticed that a higher degree of processing was positively correlated with a higher reduction of the pathogen (*p* < 0.05). 

#### 3.4.2. The Influence of the Environment and the Period of Storage 

An important factor affecting the number of *Bacillus* cells turned out to be the environmental conditions represented in three experimental variants (*p* < 0.05). Both in the sample fermented without the addition of any substances (control) and in the sample with lactic acid, the increase of *Bacillus* strains number was observed. In the sample where *L. lactis* ATCC 11454 was used, the antimicrobial effect of LAB, as well as their metabolites on decreasing viable *Bacillus* cells were demonstrated (Table 3). During storage, it was shown that the samples with lactic acid had a significantly higher ability to reduce the amount of *Bacillus* compared to the control sample (*p* < 0.05). Thus, the antimicrobial potential of the LAB metabolites, such as naturally produced organic acids as well as BLIS, may be an important bacteriostatic agent during storage and determine the inhibiting effects concerning selected *Bacillus* strains. In the research of Demirbas et al. [55], *L. lactis* has been recognized as an effective biocontrol agent against Cl. sporogenes when the inoculated sample has been stored at 4 °C. In addition, Naibaho [17] noticed that the relationship between the addition of BSG and stable production of organic acids during the fermentation and as a result decreasing pH in the fermented products could be identified as a dominant factor of the higher growth during the refrigerated storage. 

Several reports have shown that antimicrobial metabolites produced by *Lactococcus lactis* exhibit broad inhibitory properties towards species that are closely related to LAB and other unrelated spoilage and pathogenic bacteria. The stability of strains *L. lactis*, as well as the antimicrobial activity of its BLIS in the in vitro study, was described by Jawan [27]. The result of their study showed that *L. lactis* has a vast potential to be applied in the food industry, such as preparation of starter culture, functional food and probiotic products. In contrast, a probiotic strain of *Lactobacillus rhamnosus* LOCK900 used at the co-fermentation system with *Staphylococcus aureus* strains was not a significant decontamination agent when inoculated at the same time [56]. In our research, the simultaneous co-fermentation of both *L. lactis* and selected *Bacillus* strains has been applied, and the high reduction level (from 2 to 4 logs) has been observed. 

#### 3.4.3. Magnitude of Inhibition

The antimicrobial potential of BLIS against *Bacillus cereus* and *Bacillus subtilis* strains based on the results of the agar disk diffusion methods is presented in Table 4. Generally, the cell-free supernatant obtained from all of the fermented BP was characterized by the potential to inactivate the growth of *Bacillus*. The origin of CFS (obtained from fermented grain or malt or BSG) had a significant impact on its antimicrobial activity (*p* < 0.05). The degree of processing of BP was positively associated with the antimicrobial properties of obtained BLIS, thus the highest potential for biosuppression of *Bacillus* cells has been observed in CFS obtained from fermented BSG. On the other hand, the variability in the *Bacillus* strains used was also recorded. *B. subtilis* ATCC 6633 has been recognized by a highest sensitivity to the antimicrobial activity of BLIS, among other strains (*p* < 0.05). In contrast, in the agar disk diffusion method, *B. cereus* ATCC 14579 was characterized by the highest resistance to the antimicrobial properties of BLIS. Based on a standard, the amount of produced nisin was set from 40 to 200 IU g^−1^, which is in line with the EU regulation 1333/2008 [57] and the highest possible addition (4000 to 4100 IU g^−1^) and was not exceeded in any case. In the research conducted by Furuta [58], the amount of nisin produced by *L. lactis* ATCC 11454 fermented on barley by-products was estimated as 1488 IU g^−1^. Wider characteristics of the yield of nisin produced by the same strain of *L. lactis* were from 720 to 8000 IU g^−1^ [49]. 

Explanations: CSF—cell free supernatant; BSG—brewer’s spent grain. Inhibition zone [mm]: -, <1 mm; +, 1–2 mm; ++, 2–3 mm; +++, >3 mm.

**Figure 2:**
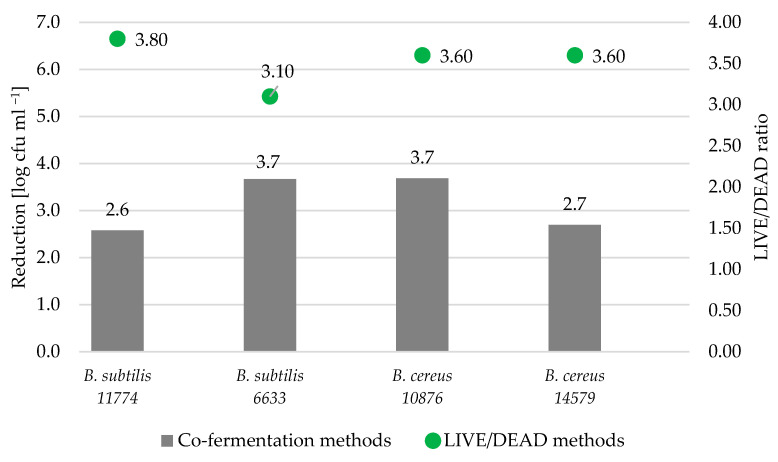
The range of log reduction of selected *Bacillus* strains after 7 days of storage and their viability under the influence of bacteriocin-like inhibitory substance (BLIS) expressed as a LIVE/DEAD ratio.

The quantitative analysis of the fluorescence ratio between the live and dead cells of *Bacillus* strains was a reliable tool to characterize the antimicrobial properties of BLIS obtained from the BSG fermented with *L. lactis* ATCC 11454, especially when the environmental condition was applied. In Figure 2, the results of viability expressed as a LIVE/DEAD ratio were juxtapositioned with the log reduction of each *Bacillus* strain after 7 days of storage. It should be noted, however, that despite the significant log reduction shown in the co-fermentation experiment (range from 2.6 to 3.7 logs), in the fluorescence analysis, the live cells of *Bacillus* were still abundant (Figure 2). Taking into account differences between strains, the most sensitive to the BLIS was the *B. subtilis* strain ATCC 6633. For which the greatest number reduction in the culture method and the lowest LIVE/DEAD ratio were observed, indicating a lower number of viable cells in the test sample compared to other tested strains.

The relationship between the spread plate and fluorescence technique has been studied by Olszewska et al. [59]. In their research, no correlation between the fluorescence analysis and the number of viable cells in the spread plate technique has been found. Moreover, significantly higher viability was usually determined based on fluorescence analysis. It is worth highlighting, that in the plate spread technique, only live and culturable cells might be determined. Fluorescence analysis could focus on cell proliferation, metabolic activity and cell structure integrity. Truchado et al. [60] observed that foodborne pathogens may maintain viability, but due to unfavorable environmental conditions, they were unable to multiply. 

In the conducted research, the adverse environmental factor, i.e., the exposition of *Bacillus* cells to the BLIS activity, was applied and the pathogen’s number decreased. This suggests that the usage of potentially antimicrobial factors could be an effective disinfection method when used in synergy. In the research of Wu et al. [61], the share of barley fermented extract (BFE) during fermentation had a significant effect on *L. lactis* ATCC 11454 cell growth, and nisin A secretion has been described. During fermentation, several metabolites are produced, such as organic acids, i.e., SCFA which due to the synergistic effect can increase the antimicrobial ability of bacteriocin in the food matrix [61]. The research conducted on different types of food products established that partially purified bacteriocin obtained from *L. lactis* C15 significantly decreases the number of viable cells of food-borne Gram-positive pathogens for over one order of magnitude compared to the control group [62]. In the research of Lee et al. [30], the nisin-produced strain of *Lactococcus lactis* 12 was used as one of the indicator strains evaluated as a potential decontamination agent. It was noticed that cell-free supernatant obtained from fermented barley β-GOS with *L. lactis* 12 has the highest potential to inactivate Gram-positive foodborne pathogens, such as *Listeria monocytogenes* KCTC 13064 *Staphylococcus aureus* subsp. *aureus* KCTC 1928, *Streptococcus iniae* KCTC 3657. In their research, the presence of purified bacteriocin weighed 3330 Da, which was structurally identical to nisin. Moreover, LAB isolated from fermented food products as *Pd. pentosaceus* BAL6 and *Pd. pentosaceus* KL14 were able to produce other antimicrobial agents, such as H_2_O_2_ [63]. Pokhrel et al. [64] noticed that biofilm could be formed by foodborne pathogens as a response to stressors or environmental factors. The novel technique of disinfection described by Kruk & Trząskowska [65], including synergetic mechanisms of chemicals (H_2_O_2_, acetic acid) and biological (a probiotic strain of *Lactobacillus plantarum* 299v), was effective in the reduction of the viable cells of the most common contamination of mung bean seeds but also prevented the formation of pathogens’ biofilm.

Moreover, it should be noted that studied experimental parameters vary from actual agricultural and environmental conditions, i.e., synergies and antagonisms exist in a more diverse environment in comparison to applied laboratory variables. Furthermore, during the laboratory experiments, optimal growth condition (time, temperature, aeration, among others) was ensured which is difficult to reach in the environment including the food chain. Also, it is well-known that many inorganic nutrients could modulate the growth of LAB and be harmful to others, such as nitrogen sources which *Lactococcus lactis* can utilize for protein synthesis and other cellular processes and for *Bacillus* could be a suppressor factor [66].

## 4. Conclusions 

The presented results indicate that processing level had a significant impact on the oligosaccharides profile and the positive correlation between the level of processing and content of both soluble and insoluble fractions of AX, and the negative correlation of β-glucans content has been reported. The fermentation process of barley products with the addition of 1% *L. lactis* ATCC 11454 increased the amount of total polyphenol content. Therefore, it indicates the potentially synergetic effect of the probiotic-candidate strain of *L. lactis* and native plant compounds, such as polyphenols, oligosaccharides as β-glucans and β-GOS. The positive correlation between the level of processing of BP used as a fermentation medium and the viability of *L. lactis* ATCC 11454 has been determined. Finally, in the in situ research was found the ability of the fermentation system consisting of post-production residues and *L. lactis* ATCC 11454 to reduce the number of selected Bacillus strains (from 2 to 4 logs). However, the fluorescence analysis indicates that the live cells of *Bacillus* have been recognized as dominant. This suggests that the cells might be transitioned into sublethal status on account of exposition to BLIS although the mechanism of inactivation of food-borne pathogens by BLIS needs to be clarified. Additionally, the impact of used BLIS on a volume and/or profile of cereulide should be thoroughly explained.

In conclusion, the obtained results justify further research on the use of brewer’s spent grains in selected food products, to assure their safety and nutritional value. This finding could be highly beneficial in the sustainable management of post-production residues when current waste material can still serve as a source of food.

## Figures and Tables

**Table 1:** Saccharides profile, arabinoxylans and beta-glucans contents in different barley products.

Saccharides		Barley Products [g kg^−1^ Dry Matter]		
		Grain	Malt	BSG
Soluble fractions of arabinoxylans (S-AX)	Arabinose	0.26	0.30	0.31
	Xylose	0.31	0.41	0.41
Total S-AX		0.57	0.71	0.72
Insoluble fractions of arabinoxylans (I-AX)	Arabinose	1.46	1.87	7.31
	Xylose	1.92	3.36	14.24
	Total I-AX	3.38	5.23	21.55
	Total AX	3.95	5.94	22.27
	Beta-glucans	4.86	0.54	0.29

Explanations: BSG—brewer’s spent grain; AX—arabinoxylans.

**Table 2:** Changes in the total polyphenols content of non-fermented or fermented barley products with Lactococcus lactis ATCC 11454 for 24 h.

Barley Product	Non-Fermented	Fermented
Grain	472 ± 26 ^Aa^	493 ± 34 ^Aa^
Malt	718 ± 36 ^Ba^	737 ± 37 ^Ba^
BSG	659 ± 27 ^Ca^	660 ± 55 ^Ca^

The result is expressed as mean value ± standard deviation [ug GAE g ^−1^ dry matter]. Explanations: BSG—brewer’s spent grain; means followed by different capital letters or lowercase in the same columns or row, respectively, present significant differences (*p* < 0.05).

**Table 3:** The mean number of selected *Bacillus* strains depending on barley products and antimicrobial agents i.e., lactic acid or *L. lactis* 11454 [log CFU g^−1^].

				Antimicrobial Agent					
		Variant I Control		Variant II Lactic Acid		Variant III Fermentation with *L. lactis* 11454			
	Day of storage	0	7	0	7	0	7	0	7
	Number of	*B. subtilis* ATCC 11774				*B. subtilis* ATCC 11774		*L. lactis* 11454	
Barley products	Grain	8.67 ^A^	7.33 ^A^	8.63 ^A^	7.48 ^A^	5.25 ^A^	3.42 ^A^	7.25 ^A^	7.87 ^A^
	Malt	8.67 ^A^	6.34 ^B^	8.70 ^A^	5.49 ^B^	4.55 ^A^	3.27 ^A^	8.01 ^B^	7.50 ^B^
	BSG	7.94 ^B^	6.61 ^B^	8.16 ^B^	6.46 ^C^	3.39 ^B^	4.42 ^AB^	6.81 ^AC^	8.38 ^C^
	Number of	*B. subtilis* ATCC 6633				*B. subtilis* ATCC 6633		*L. lactis* 11454	
Barley products	Grain	7.78 ^A^	5.35 ^A^	7.70 ^A^	5.47 ^A^	3.80 ^A^	3.05 ^A^	7.90 ^A^	8.05 ^A^
	Malt	7.66 ^A^	8.14 ^B^	7.97 ^A^	4.98 ^A^	3.27 ^A^	4.20 ^B^	8.12 ^B^	7.66 ^A^
	BSG	6.89 ^B^	5.41 ^AC^	6.96 ^AB^	4.61 ^AB^	3.24 ^AB^	3.33 ^C^	8.03 ^AC^	7.96 ^A^
	Number of	*B. cereus* ATCC 10876				*B. cereus* ATCC 10876		*L. lactis* 11454	
Barley products	Grain	8.22 ^A^	7.23 ^A^	7.81 ^A^	6.42 ^A^	4.67 ^A^	4.33 ^A^	7.88 ^A^	7.76 ^A^
	Malt	7.77 ^B^	6.81 ^A^	7.85 ^A^	5.31 ^B^	4.41 ^A^	3.74 ^A^	7.96 ^B^	7.83 ^B^
	BSG	8.00 ^AB^	6.98 ^A^	6.35 ^B^	5.53 ^B^	3.97 ^AB^	3.32 ^AB^	8.17 ^C^	8.19 ^C^
	Number of	*B. cereus* ATCC 14579				*B. cereus* ATCC 14579		*L. lactis* 11454	
Barley products	Grain	7.78 ^A^	6.31 ^A^	7.72 ^A^	5.65 ^A^	3.83 ^A^	4.07 ^A^	8.05 ^A^	7.60 ^A^
	Malt	7.53 ^B^	5.27 ^B^	7.70 ^A^	5.10 ^A^	4.51 ^B^	3.30 ^A^	8.05 ^A^	8.03 ^B^
	BSG	6.63 ^C^	6.25 ^AC^	7.71 ^A^	6.32 ^AB^	4.91 ^C^	4.30 ^AB^	8.41 ^A^	8.32 ^B^

Explanation: BSG—brewer’s spent grain; control—no antimicrobial agents were used; means in the same columns between different rows followed by different capital letters represent significant differences (*p* < 0.05).

**Table 4:** Antimicrobial activity of CFS from different media fermented with L. lactis ATCC 11454 (determined by the agar well diffusion assay).

	*Bacillus* Strain			
CFS–Medium	*B. subtilis* ATCC 11774	*B. subtilis* ATCC 6633	*B. cereus* ATCC 10876	*B. cereus* ATCC 14579
Grain	++	++	+	+
Malt	++	++	+++	-
BSG	+	+++	++	++

Explanations: CSF—cell free supernatant; BSG—brewer’s spent grain. Inhibition zone [mm]: -, <1 mm; +, 1–2 mm; ++, 2–3 mm; +++, >3 mm.

## Data Availability

The data used to support the findings of this study can be made available by the corresponding author upon request.
